# Genome-Wide Characterization and Expression of Two-Component System Genes in Cytokinin-Regulated Gall Formation in *Zizania latifolia*

**DOI:** 10.3390/plants9111409

**Published:** 2020-10-22

**Authors:** Lili He, Feng Zhang, Xiaozhen Wu, Yanmei Hu, LiLi Dong, Walter Dewitte, Bo Wen

**Affiliations:** 1 School of Horticulture, Anhui Agricultural University, Hefei 230036, China; HLily@ahau.edu.cn (L.H.); zhangfeng0801@ahau.edu.cn (F.Z.); xiaozhen.wu@nonglesw.com (X.W.); auhym@ahau.edu.cn (Y.H.); dongli0608@163.com (L.D.); 2 School of Biosciences, Cardiff University, Museum Avenue, Cardiff CF10 3AX, UK

**Keywords:** two-component system, *Z. latifolia*, shoot swelling, cytokinin signals

## Abstract

The thickening of *Zizania latifolia* shoots, referred to as gall formation, depends on infection with the fungal endophyte *Ustilago esculenta*. The swollen and juicy shoots are a popular vegetable in Asia. A key role for cytokinin action in this process was postulated. Here, trans-zeatin stimulated swelling in fungi-infected *Z. latifolia*. A two-component system (TCS) linked cytokinin binding to receptors with transcriptional regulation in the nucleus and played important roles in diverse biological processes. We characterized 69 TCS genes encoding for 25 histidine kinase/histidine-kinase-like (HK(L)) (21 HKs and 4 HKLs), 8 histidine phosphotransfer proteins (HP) (5 authentic and 3 pseudo), and 36 response regulators (RR; 14 type A, 14 type B, 2 type C, and 6 pseudo) in the genome of *Z. latifolia*. These TCS genes have a close phylogenetic relationship with their rice counterparts. Nineteen duplicated TCS gene pairs were found and the ratio of nonsynonymous to synonymous mutations indicated that a strong purifying selection acted on these duplicated genes, leading to few mutations during evolution. Finally, *ZlCHK1*, *ZlRRA5*, *ZIRRA9*, *ZlRRA10*, *ZlPRR1*, and *ZlPHYA* expression was associated with gall formation. Among them, *ARR5*, *ARR9*, and *ZlPHYA* are quickly induced by trans-zeatin, suggesting a role for cytokinin signaling in shoot swelling of *Z. latifolia*.

## 1. Introduction

*Zizania latifolia* belongs to the wild rice genus *Zizania* and was an important grain crop in ancient China. Around 2000 years ago, *Z. latifolia* was infected by the fungal endophyte *Ustilago esculenta* which causes swelling in shoot apical tissues, resulting in juicy galls. Nowadays, the swollen shoots of *Z. latifolia* are a popular vegetable in China (called “Jiaobai”), East Asian countries, and Japan [1,2].

In nature, plant tumors or “galls” can be induced by different pathogens such as bacteria, viruses, fungi, protists, and insects. Despite different pathogenic mechanism to induce galls, most of these plant pathogens demonstrate the ability to change the phytohormone level in the host plants, especially cytokinins. For example, the well-studied gall-forming *Agrobacterium tumefaciens* carries the *Ti* plasmid, which can integrate a DNA segment called T-DNA into the host’s genome [3]. The T-DNA carries several genes including isopentenyl transferase (IPT), a key enzyme of cytokinin biosynthesis, which instigates increased cytokinin production in the transformed cells of the host [4].

Increased production of cytokinins in the infected plant leads to uncontrolled cell proliferation and gall formation [5,6]. Chan and Thrower [7] isolated zeatin in *Ustilago esculenta* liquid culture, suggesting this fungi may also produce cytokinin in vivo. Another study revealed that the content of cis-zeatin riboside and cytokinin activity in terms of kinetin equivalents from fungi-infected gall tissue was elevated compared to non-fungi-infected shoots in *Z. latifolia* [8]. These results indicate that cytokinins play an important role in the swelling of *Z. latifolia* shoots.

Cytokinins are a class of key phytohormones that mediate several processes including apical dominance, lateral branching, germination, flower and fruit development, chloroplast differentiation, plant–pathogen interactions, senescence, cell division in shoots, and spatial control of differentiation in roots [9,10,11,12,13]. Cytokinin signal transduction is through a phospho-relay derived from the two-component system (TCS) consisting of membrane-associated histidine kinase receptor proteins (HK), histidine-containing phosphotransfer proteins (HPs), and the response regulators (RR) gene. The TCS genes in the *Arabidopsis thaliana* model have been identified and functionally characterized. Three histidine kinase proteins of AHK2, AHK3, and AHK4 have been characterized as cytokinin receptors [14]. All cytokinin receptors were found to share a domain in the predicted extracytoplasmic region, designated as cyclase/histidine kinase-associated sensory extracellular (CHASE) domain which is the putative binding site for cytokinin molecules [15]. Upon perceiving the stimuli, the receptors are autophosphorylated. The phosphate group is then transferred to the AHPs (Arabidopsis histidine-containing phosphotransfer proteins), which are able to enter the nucleus and transfer the phosphate group to type B RRs. In the nucleus, phosphorylated type B RRs can, amongst other targets, transcriptionally activate type A RRs. Type A RRs act as repressors of the primary cytokinin response, providing a negative feedback regulation [16,17,18]. This TCS-mediated histidine-aspartate signaling has been demonstrated to be able to control diverse biological processes, such as osmosensing, responses to environmental stimuli, cell growth, and proliferation [14,19,20,21,22,23,24,25,26,27,28,29,30]

Capitalizing on the progress in genome sequencing, TCS genes have been identified and characterized in many other different plant species such as rice [21], maize [25], soybean [24], Chinese cabbage [26], tomato [27], and cucumber [28]. In this study, the effect of exogenous trans-zeatin in *Z. latifolia* shoots swelling was investigated. Exogenous cytokinin stimulated the gall formation in fungi-infected shoots, in line with previous reports. As a first step towards understanding this response to cytokinins, genome-wide identification of TCS genes in *Z. latifolia* was conducted, which included sequences and functional domains analysis, phylogenetic relationship prediction, genes and protein structure analysis, and the analysis of the evolution of these members. Secondly, the expression of the TCS genes during shoot swelling was investigated by transcriptomics. Thirdly, the response of a subset of TCS genes to exogenous cytokinin was analyzed by quantitative real-time PCR (qRT-PCR). The obtained results establish a base for future functional analysis of TCS genes in cytokinin signal transduction in *Z. latifolia*, especially during gall formation.

## 2. Results and Discussion

### 2.1. Effect of Exogenous Cytokinin Treatment on Z. latifolia Gall Formation

**Figure 1:**
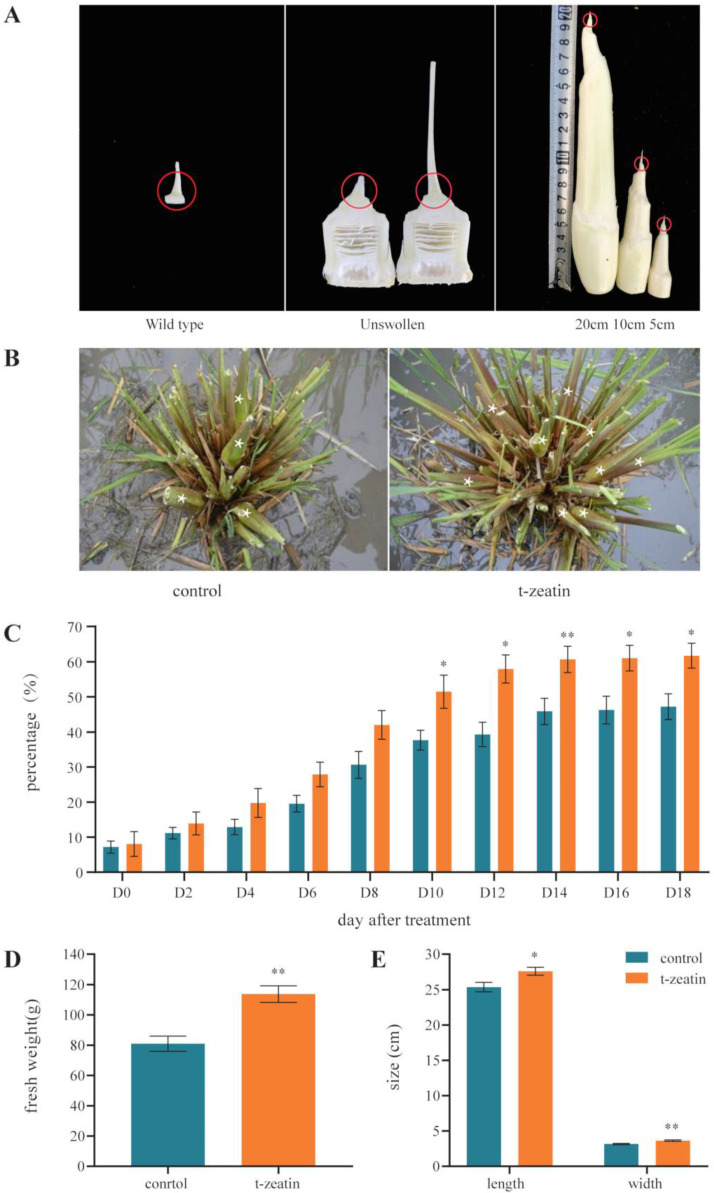
Development of and effects of cytokinin on *Zizania latifolia* galls. (**A**) In contrast to uninfected wild-type (WT) shoots (left panel), infected shoots develop large galls under their apices up to 20 cm length, over a time course of 2 weeks (middle, initial shape of apex, right panel stages of gall formation). The red circles indicate the size of the collected samples collected for expression analysis. (**B**) Morphology (stars label the swollen shoots) and (**C**) frequency of gall formation of 150 mg/L t-zeatin treated and untreated *Z. latifolia* plants (*n* = 12). (**D**) Fresh weight and size (**E**) of treated and untreated galls (*n* > 100). Each histogram represents the mean value and the bar indicates the means ± standard error of biological replicates. * Significant difference at *p* < 0.05. ** Significant difference at *p* < 0.01.

In contrast to non-infected wild-type *Z. latifolia* shoots, *Ustilago esculenta*-infected plants develop galls which become up to 20 cm long over two weeks (Figure 1A). Trans-zeatin treatment could significantly increase the frequency of shoot swelling by about 15% (*p* < 0.05) (Figure 1B,C). Further measurements suggested that cytokinin treatment also significantly stimulated the shoots swelling in size and weight (*p* < 0.05) (Figure 1D,E). Chan and Thrower [7] suggested that three kinds of cytokinins including zeatin and zeatin riboside were isolated in *Z. latifolia* gall tissue, and *Ustilago esculenta* could produce zeatin in culture. However, no structural confirmation on the cytokinin identity was available at time. A higher level of cis-zeatin riboside was detected in the *Z. latifolia* gall compared to the uninfected shoot [8]. Furthermore, *ZlIPT*, the most important enzyme to catalyze cytokinins synthesis, was more highly expressed in gall tissue compared to the uninfected shoot [31]. Collectively, cytokinins may play a key role in *Z. latifolia* gall formation and the cytokinins produced by *Z. latifolia* and/or *Ustilago esculenta* could stimulate cell division and enlargement, facilitating gall formation. This study also demonstrated that exogenous cytokinin could stimulate and promote the gall formation; therefore, cytokinins can be used as a plant growth regulator to improve the horticultural production of *Z. latifolia* galls.

### 2.2. Identification of TCS Genes in Z. latifolia

To identify members of the TCS family in *Z. latifolia*, HMM searches and BLASTP analysis were performed by employing 415 TCS protein sequences from *Arabidopsis* [14], rice [21], maize [25], soybean [24], Chinese cabbage [26], and tomato [27] as queries. After repetitive sequences were removed, all identified sequences were reserved and submitted to NCBI CDD, Pfam, and SMART to confirm their typical domains. A total of 69 non-redundant sequences, including 25 HK(L)s, 8 HPs, and 36 RRs, were identified in the *Z. latifolia* genome. All TCS members in *Z. latifolia* were named according to the homologous genes in *Arabidopsis* [27,28,32]. This nomenclature has been used in tomato [27], cucumber, and watermelon [28]. The TCS genes have been intensively studied in model plants and some important crops. The number of TCS genes in *Z. latifolia* (*n* = 69) is similar to that in tomato (*n* = 65) but larger than that of all reported species except soybean (*n* = 98) and Chinese cabbage (*n* = 85; Table S1).

### 2.3. HK Proteins in Z. latifolia

Sequence analysis of the entire *Z. latifolia* genome revealed 25 distinct putative histidine protein kinases genes, including 21 histidine protein kinases (ZlHKs) and 4 related proteins (ZlHKLs), based on whether their HK domains were conserved or divergent (Table 1). These histidine protein kinase homologs can be further divided into distinct subfamilies: Eight cytokinin receptors (CHKs), one CKI-like protein, seven ethylene receptors (ERSs and ETRs), five phytochrome photoreceptors (PHYs), and four pyruvate dehydrogenase kinases (PDKs; Table 1). A typical HK domain (corresponding to motifs 1 and 7 in Figure S2) has five conserved signature motifs—H, N, G1, F, and G2—with the conserved His as the key feature in the H motif. The other four motifs were defined as the nucleotide-binding cleft [33]. Through multiple-sequence alignment, all HKs and HKLs proteins were found to be conserved in the H motif with His residue, except ZlCHK2 and ZlCHK3 (Figure S1A). All proteins had complete N motifs. However, the G1, F, and G2 motifs occurred only in ZlCHK3 and ZlCHK4, suggesting that other HK/HKL proteins may not have full HK function or that these motifs may not be necessary for HK function (Figure S1A). Notably, only ZlCHK4 had a full HK domain, suggesting that it has a critical function in *Z. latifolia*. Table 1 and Figure S1B also show that both CHASE domains (motif 11 in Figure S2) and 1–4 transmembrane sequences (TM) occurred in these cytokinin receptors, except for ZlCHK2 and ZlCHK3. Both CHASE and TM were demonstrated to be crucial for membrane-associated cytokinin recognizing and binding [17]. In addition, the receiver domain (motifs 9 and 13 in Figure S2) with Asp residue was conserved in all ZlCHKs except for ZlCHK7 (Figure S1B). Two AHK2-like genes (ZlCHK1 and ZlCHK2) were 65% and 46% similar, respectively, to their *Arabidopsis* counterparts of AHK2, whereas two AHK3-like members (ZlCHK3 and ZlCHK4) were 61% and 63% similar, respectively, to *Arabidopsis* AHK3. Another four AHK4-like cytokinin receptor genes (ZlCHK5, ZlCHK6, ZlCHK7, and ZlCHK8) were highly similar (55%–72%) to *Arabidopsis* CRE1/AHK4/WOL (Table 1). AHK2, AHK3, and CRE1/AHK4/WOL are cytokinin receptors and can regulate cell division in *Arabidopsis* [15,34,35,36,37,38,39,40,41]. Some of the cell cycle and meristem control master genes, such as *CYCD3s* and *STM1*, respond to cytokinins and function downstream of CRE1/AHK4/WOL to regulate shoot and root development [42].

The ethylene receptors, including ZlETR1–4 and ZlERS1–3, contain HK, HATPase, and cyclic GMP adenylyl cyclase FhlA (GAF) domains (Table 1). Although histidine–kinase activity can subtly modulate the ethylene response, no major role has yet been identified in ethylene signal transduction [43,44,45]. However, histidine kinase activity could allow cross-talk between ethylene perception and other TCS pathways such as cytokinin signal transduction. All five PHY subfamily members (ZlPHYA, B, C, D, E) possess PHY (chromophore-binding), GAF, and PAS (signal sensor) domains (Table 1). These domains were found to be essential for responding to red and far-red light signals during plant development in *Arabidopsis* [46]. The HATPase domain was identified in all ZlPDKs proteins (ZlPDK1–4). A newly published paper has revealed that PDK1 could regulate auxin transport and vascular development through phosphorylation of AGC1 kinase PAX in *Arabidopsis* [47].

To further understand the structural diversity of *Z. latifolia* HK genes, we analyzed the exon–intron organization and conserved protein motifs (Figure S2). These analyses revealed that HK genes in the same group usually had a similar gene structure and motif composition, which strongly support the reliability of the phylogenetic classification.

### 2.4. HP Proteins in Z. latifolia

Eight ZlHP genes were identified: Five authentic HPs and three pseudo-HPs (Table 2). All ZlHPs were small proteins with 149–276 amino acids and had two conserved motifs (motifs 1 and 2 in Figure S3) that encode the Hpt domain. Five authentic HPs (ZlHP1, ZlHP2, ZlHP4, ZlHP7, ZlHP8) contained conserved His (H) residues in the HP signature sequence of XHQXKGSSXS, whereas in the other three pseudo-HPs (ZlHP3, ZlHP5, and ZlHP6), the histidine phosphorylation site was replaced by Gln (Q) residue (Figure S4). Notably, all HPs except ZlHP1 and ZlHP3 were localized in the nucleus (Table 2), which might be essential for their phosphorelay, during which HPs translocate from the cytoplasm to the nucleus [48,49]. In addition, all ZlHPs had a very similar gene structure containing 5–7 introns and motif compositions (Figure S3).

Two AHP1-like members of ZlHP1 and ZlHP2 were 20% and 50% similar to their *Arabidopsis* homolog AHP1, respectively (Table 2). AHP1 could interact with CRE1/AHK4/WOL and B-type ARRs such as ARR1, suggesting that it may function as a phosphotransfer intermediate [50,51,52,53]. Four AHP4-like members (ZlHP3, ZlHP4, ZlHP5, ZlHP6) were found to be highly similar (59%–63%) to *Arabidopsis* AHP4 (Table 2). Interestingly, three pseudo-HPs of (ZlHP3, ZlHP5, ZlHP6) had a higher similarity to the authentic AHP4 than to the pseudo APHP1 in *Arabidopsis*. This was also observed in rice and cucumber [21,28]. Both ZlHP7 and ZlHP8 were 46% similar to their *Arabidopsis* counterpart AHP5 (Table 2). The high sequence similarity between ZlHP7 and ZlHP8 suggests possible functional redundancy between these two genes.

### 2.5. RR Proteins in Z. latifolia

Thirty-six genes were identified as ZlRRs: 14 type A RRs, 14 type B RRs, 2 type C RRs, and 6 pseudo-RRs (Table 3). The type A RRs (ZlRRA1–14) were a group of small proteins with 76–269 amino acids. All type A RRs contained a receiver domain corresponding to three motifs (motifs 1, 3, and 4 in Figure S5) along with short N- and C-terminal extensions (Table 3 and Figure S5). Most type A RRs contained four introns. The 14 ZlRRAs were highly similar (52%–81%) to their *Arabidopsis* homologs (Table 3).

In addition to having a conserved receiver domain, all type B RRs (ZlRRB1–14) contained long C-terminal extensions with a Myb-like DNA binding domain (motif 2 in Figure S5), suggesting their function as transcription factors. Except for ZlRRB6, all type B RRs were predicted to be localized in the nucleus (Table 3). Most type B RRs had four or five introns (Figure S5). Type B RRs were 30%–66% similar to their counterparts in *Arabidopsis*.

Different from type A RRs, two identified type C RRs (ZlRRC1, 2) contained a receiver domain (motifs 1 and 4 in Figure S5) but lacked a long C-terminal extension. Both proteins had three introns and were 40% and 41% similar to their *Arabidopsis* counterpart, ARR24, respectively. In addition, six proteins identified as pseudo-RRs (ZlPRR1–6) all had a conserved receiver domain (motifs 1 and 4 in Figure S5) and a CCT domain (motif 5), which was found to play an important role in regulating circadian rhythms [54]. These pseudo-RRs were 39%–54% similar to their counterparts in *Arabidopsis*. Except ZlPRR2, all these type C and pseudo-RRs were localized in the nucleus (Table 3, Figure S5). Figure S5 also shows that the ZlRRs genes in the same category usually had very similar gene structure and motif composition, thereby supporting the evolutionary conservation and reliability of the phylogenetic classification.

### 2.6. Phylogenetic Relationship of TCS Members in Z. latifolia

**Figure 2:**
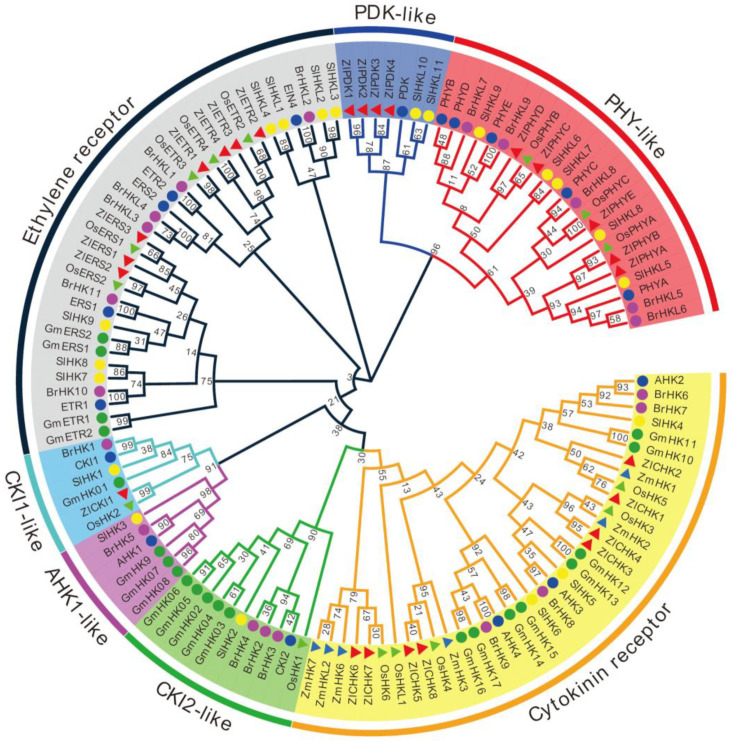
Phylogenetic relationship of HK(L) proteins in *Arabidopsis*, rice, maize, soybean, Chinese cabbage, tomato, and *Z. latifolia.* A maximum likelihood phylogenetic tree indicates the different subgroups of HK(L)s in different colors.

To investigate the evolutionary relationships of TCS genes, we used all amino acid sequences of 124 HK(L)s, 53 HPs, and 275 RRs from *Arabidopsis* [14], rice [21], maize [25], soybean [24], Chinese cabbage [26], tomato [27], and *Z. latifolia* to construct maximum likelihood (ML) trees [55]. On the basis of the bootstrap values and the topology of the tree, all HK(L)s proteins in the seven species were divided into seven distinct subfamilies, including cytokinin receptor, ethylene receptor, PHY-like, CKI1-like, CKI2/AHK5-like, AHK1-like, and PDK-like subfamilies (Figure 2). Similar phylogenetic structures were viewed in previous studies [14,17,21]. The HK(L)s in *Z. latifolia* usually have much closer relationships to those of rice and maize than other species, as they are all members of the monocotyledon. This indicates that the HK(L)s gene expansion occurred after the divergence of monocot and dicot plants. In addition, HK(L)s genes of monocots and eudicots showed an alternative distribution pattern in each subfamily, but no monocots occurred in the AHK1-like subgroup.

**Figure 3:**
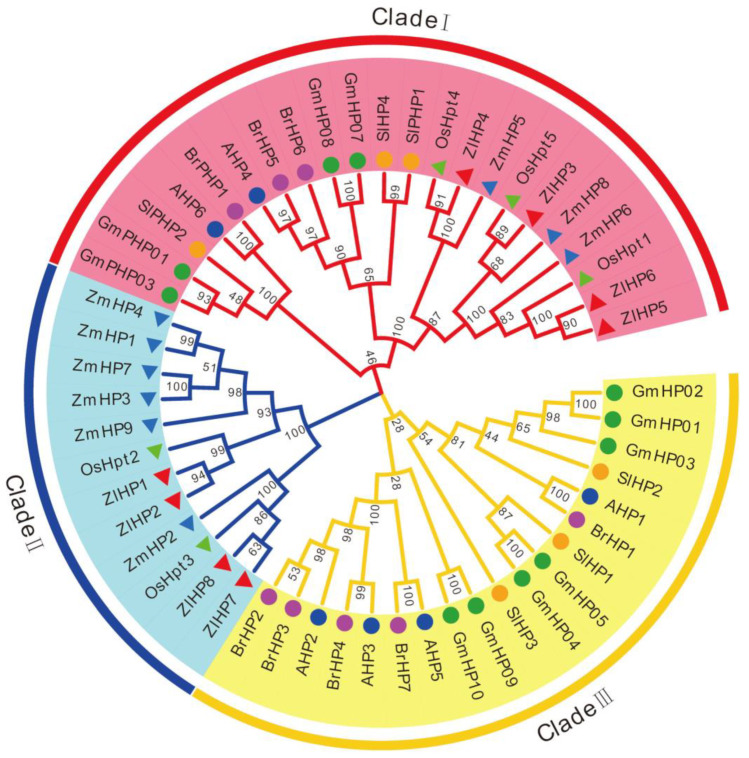
Phylogenetic relationship of phosphotransfer proteins (HP) and related proteins in *Arabidopsis*, rice, maize, soybean, Chinese cabbage, tomato, and *Z. latifolia*.

All HP proteins from the seven species were divided into three clades—clades I, II, and III (Figure 3). Clade II contained HPs only from monocots, whereas all HPs in clade III were from dicots. These results suggest that the gene expansion events for HPs occurred after the divergence of monocots and dicots. Four authentic ZlHPs except ZlHP4 were grouped into clade II, whereas clade I mainly contained pseudo-HPs from both monocots and dicots. All ZlHPs showed the closest phylogenetic relationship to rice OsHpts (Figure 3).

**Figure 4:**
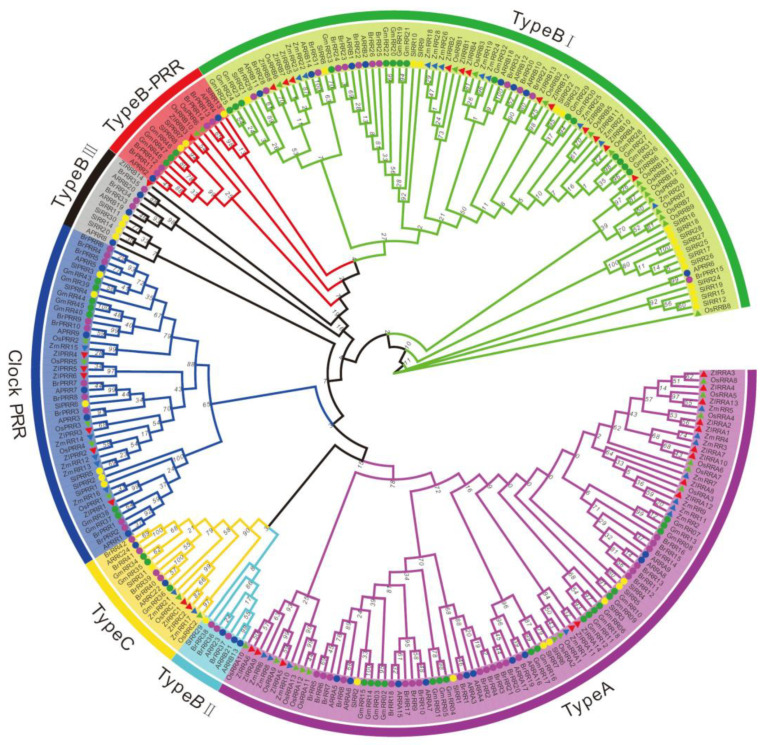
Phylogenetic relationship of response regulator (RR) proteins and related proteins in *Arabidopsis*, rice, maize, soybean, Chinese cabbage, tomato, and *Z. latifolia*.

The 275 RR proteins from the seven plant species were classified into type A, type B, type C, and pseudo-RR groups (Figure 4). Generally, the ZlRRs in *Z. latifolia* were phylogenetically closer to rice OsRRs in all these groups. Phylogenetic analyses indicated that all type A RRs shared a close evolutionary relationship and an alternating distribution between monocots and dicots plants, suggesting that type A RR genes might already have expanded before the monocot–dicot divergence. Type B RRs from these species could be divided into three subgroups (I, II, and III), consistent with previous studies [17,26]. In detail, type B II subgroups contain RRs only from dicots of *Arabidopsis*, Chinese cabbage, and tomato; the RRs from monocots might have been lost during evolution. All type B ZlRRs from *Z. latifolia* except for ZlRRB3 and ZlRRB14 were classified into the type B I subfamily. A similar phenomenon was also viewed in soybean and cucumber [24,28]. All pseudo-RRs from seven plant species were subclassified into type B PRRs or clock PRRs (Figure 4).

### 2.7. Strong Purifying Selection for TCS Genes in Z. latifolia

**Figure 5:**
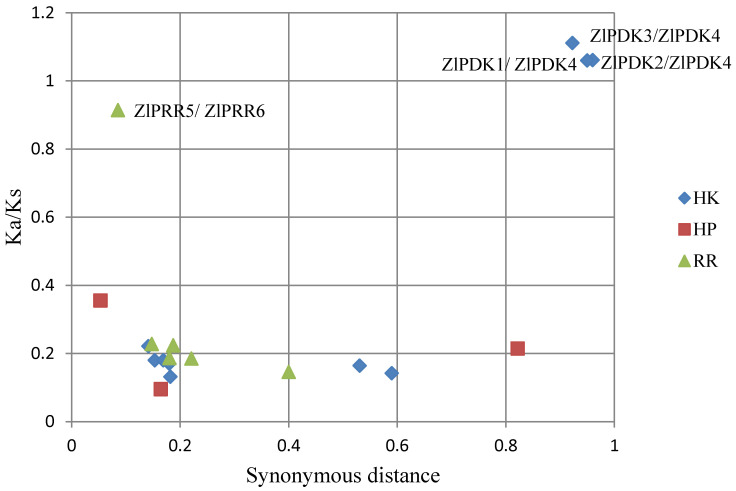
Scatter plots of the Ka/Ks ratios of duplicated two-component system (TCS) genes in *Z. latifolia*. The x-axis represents the synonymous distance and the y-axis represents the Ka/Ks ratio for each pair.

To further understand how the TCS gene family in *Z. latifolia* expanded during evolution, the gene duplication events were investigated. Nineteen duplicated gene pairs in *Z. latifolia* TCS genes were identified including 10 ZlHK pairs, 3 ZlHP pairs, and 6 ZlRR pairs (Table S2). To study the selection pressures on the TCS gene family, nonsynonymous (Ka), synonymous (Ks), and Ka/Ks ratios were calculated. In this study, the Ka/Ks values from the 19 pairs of *Z. latifolia* TCS genes were much less than 1, except *ZlPDK1*/*ZlPDK4, ZlPDK2*/*ZlPDK4*, and *ZlPDK3/ZlPDK4* (Table S2, Figure 5). This suggests that the TCS gene family in *Z. latifolia* underwent strong purifying selection, with a slow rate of accumulation of missense mutations during evolution.

### 2.8. TCS Gene Expression During Z. latifolia Shoots Swelling

To gain more insight into the TCS gene function in cytokinin-regulated gall formation, the transcriptome measured by RNA sequencing was mined to investigate the expression of TCS genes in shoot apices during shoot swelling in *Z. latifolia*. Levels of transcripts of TCS genes in wild-type (collected from uninfected shoots) were compared to samples from young but infected unswollen shoots (collected from fungi-infected plant with eight leaves) and samples from developing galls (Figure 1A).

**Figure 6:**
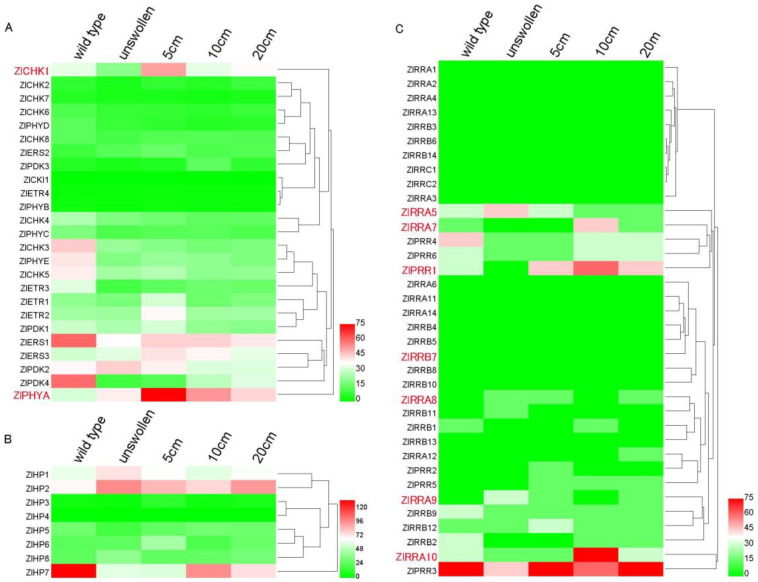
Heatmap showing the expression of ZlHK(L)s (**A**), ZlHPs (**B**), and ZlRRs (**C**) in *Z. latifolia*. The expression levels of genes are presented using transcripts Per Million reads (TPM) values and the color scale are shown at the top of heat map. Green represents low expression and red represents strong expression.

We noted the transient kinetics of the *ZICHK1* and *ZIETR2* receptor transcripts, the transcripts of the gene encoding for the red-light receptor phytochrome A (*ZIPHA*) transiently accumulated during gall formation and the activation of *ZIPDK2* in young infected shoots (Figure 6A). Elevated expression of the phosphotransfer protein *ZIAHP1*, *ZIAHP2*, and *ZIAHP6* was associated with infected shoots (Figure 6B). We noted that the homologous gene pairs of *ZlHP2/ZlHP7* showed different expression patterns, suggesting their functional divergence after the duplication event (Figure 6B). The response regulator encoding *ZlRRA5* and *ZIARR9* was activated early, whereas *ZIAAR7* and *ZlRRA10* type A and *ZlPRR1* response regulator genes were activated later in more developed galls. Overall, levels of ARRB type encoding transcripts were lower, with *ZIRRB12* displaying a higher level of transient expression kinetics (Figure 6C). To validate the transcriptome data, the transcript levels of five genes with distinct kinetics including an HK, HPs, and an RR and PHYA were monitored by qPCR. The expression trends agreed with the RNA seq data, suggesting the RNA seq results were reliable (Figure S6).

### 2.9. TCS Gene Response to Exogenous Cytokinin

**Figure 7:**
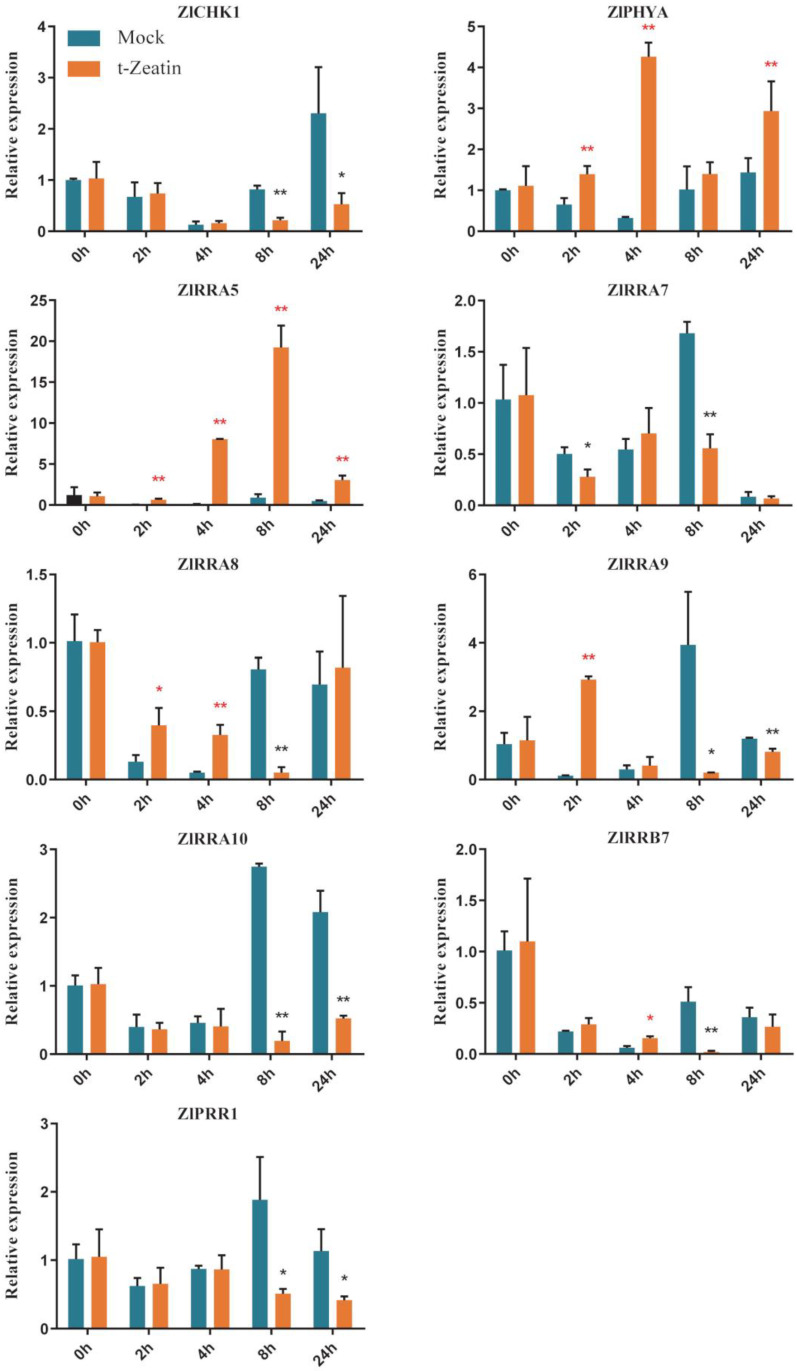
Expression kinetics of nine selected TCS genes in response to trans-zeatin treatment in *Z. latifolia*. Data are the means ± standard error (*n* = 3). Two percent EtOH was used as mock treatment. * Indicates the significant differences (*p* < 0.05) and ** indicates significant differences (*p* < 0.01).

Our observations indicated that exogenous cytokinin can increase the frequency of gall formation and promote the growth of galls in *Z. latifolia* shoots. To verify whether the gall-associated TCS genes were responsive to exogenous cytokinin, the expression of 9 selected genes was measured through real-time PCR upon trans-zeatin treatment (Figure 7). Transcript levels of *ZlCHK1*, *ZIRRA5*, *ZlRRA7*, *ZIRRA8*, *ZIRRA9*, *ZlRRA10*, *ZIPHYA, ZIRRB7*, and pseudo-RR gene of *ZlPRR1* in samples of 150 mg/L trans-zeatin sprayed shoots were compared with mock-sprayed samples after 0 (9:00 am in the morning), 2, 4, 8, and 24 h. The mock-sprayed samples displayed a similar trend for all genes measured. In these mock samples, we observed a transient decline in transcript levels followed by an increase after 8 h. In contrast, transcript levels of *ZIRRA5*, *ZIRRA9*, and *ZIPHYA* were elevated by zeatin treatment after two to four hours (Figure 7), and this response was transient. These results indicate that the expression of only a subset of A-type RR genes is triggered quite rapidly upon cytokinin treatment, indicating the complexity of cytokinin signaling-regulated *Z. latifolia* gall formation. Interestingly, the expression of red and far-red light sensor genes of *ZlPHYA* was quickly induced after 4 h trans-zeatin treatment (Figure 7). In *Arabidopsis*, *PHYA*, the counterpart gene of *ZlPHYA* played various roles in light-regulated plant development such as seed germination [56,57], internode elongation [58], and root hair growth [59]. The function of *ZlPHYA* in cytokinin-regulated gall swelling in *Z. latifolia* needs further elucidation.

## 3. Materials and Methods

### 3.1. Plant Growth and Treatments

*Z. latifolia* cv. Ivory, was provided by Zibing Ge, Shucheng Agricultural Science Research Institute, Demonstration Park of Agricultural Science Institute of Taoxi Town, Shucheng, China. The plants were grown in the field of the Anhui Agricultural University Farm (Hefei, China). To investigate the morphological changes of *Z. latifolia* in response to cytokinin, a 150 mg/L trans-zeatin (HENUO WEIYE Co., China) solution in 2% EtOH was sprayed onto the leaves of fungi-infected plants two weeks before their shoot swelling began. Two percent EtOH was used as mock treatment. The frequency of swelling was recorded daily over a period of 18 days. As the swelling ceased, shoots were harvested, and weight and size were recorded.

To examine the expression profiles of TCS genes responding to cytokinin, 150 mg/L trans-zeatin solution was sprayed until the fully expanded leaves were all covered with the solution. The shoots’ apical tissues were then collected at 0, 1, 2, 4, 8, and 24 h after treatments. All samples were collected in three biological replicates with 3 shoots each and then stored at −80 °C until RNA extraction.

### 3.2. Identification of TCS Genes in Z. latifolia

The genome sequences of *Z. latifolia* were downloaded from the Ricerelativesb GD (http://ibi.zju.edu.cn/ricerelativesgd/download.php). The protein sequences of other TCS genes from *Arabidopsis*, rice, maize, tomato, soybean, and Chinese cabbage were downloaded from Phytozome (http://phytozome.jgi.doe.gov/pz/portal.html) or NCBI (www.ncbi.nlm.nih.gov). All TCS sequences were used to build a local blast database and then used as queries to perform BLASTP (Basic Local Alignment Search Tool: Protein BLAST) searches with an E-value of 1e^−5^ as the threshold [24,60]. However, the putative TCS proteins of *Z. latifolia* were searched with the hidden Markov model (HMM) of TCS characteristic domains from Pfam (http://pfam.janelia.org/). On the basis of the above two independent methods, the redundancy removal sequences were obtained. To confirm the reliability of the searched results, all candidate protein sequences were then confirmed with SMART (http://smart.embl-heidelberg.de/) [61], Pfam (http://pfam.xfam.org/) [62,63], and NCBI Conserved Domain Database (CDD) (https://www.ncbi.nlm.nih.gov/cdd/) [64] according to whether these sequences possess the specific structural characteristics and conserved domains of TCS elements, including HisKA, HATPase, CHASE, His-containing phosphotransfer (HPt), and receiver (Rec) domains.

### 3.3. Genes Structure Prediction, Protein Sequence Analysis, and Phylogenetic Analysis

Analysis of exons and introns were conducted using Gene Structure Display Server 2.04 (http://gsds.cbi.pku.edu.cn/) [65] by comparing the coding sequences with their corresponding gene sequences. The isoelectric point (*PI*) and protein molecular weight in kDa were obtained using ExPASy proteomics server (http://www.expasy.org/tools/) [66]. Protein subcellular localizations were predicted using Target P online software (http://www.cbs.dtu.dk/services/TargetP/). Multiple Expectation Maximization for Motif Elicitation (MEME; http://meme.sdsc.edu/meme4_3_0/intro.html) [67] was used for motif analysis to confirm the presence of the conserved motifs in these TCS proteins. Multiple-sequence alignment for the predicted peptide sequences of conserved domains was generated using default parameters [68]. Next, phylogenetic analysis was performed with the MEGA 7 program by using the maximum likelihood (ML) method with 1000 replicates of the bootstrap based on the full-length protein sequences [69].

### 3.4. Calculation of Nonsynonymous (Ka) to Synonymous (Ks) Substitutions

The TCS paralogous genes in *Z. latifolia* were subjected to a manual BLAST search, and two genes with a similarity of >75% were considered paralogous gene pairs. However, the tandem or segmental duplication genes could not be determined due to the lack of chromosome information. The selection pressure of paralogs was then determined by analyzing the synonymous and nonsynonymous nucleotide substitutions rates (Ka/Ks ratio) of each paralogous gene pair. In theory, Ka/Ks < 1 indicates purifying or negative selection, Ka/Ks = 1 indicates neutral selection, and Ka/Ks > 1 indicates positive selection [70]. The Ka/Ks ratio was calculated using DnaSP v 5.0 software [71].

### 3.5. Transcriptome Analysis and Expression of TCS Genes

To obtain a better understanding of the functions of the TCS genes, we investigated their transcriptional levels in the different gall formation stages in *Z. latifolia*. Samples from shoots apical tissue (0.5 cm in length) were collected from wild-type *Z. latifolia* (no fungi infected and never swollen), and galls in different size including non-swollen, 5, 10, and 20 cm, respectively. All samples were collected in three biological replicates with 3 shoots each, frozen immediately in liquid nitrogen, and then stored at −80 °C until used. RNA was extracted and the cDNA library was constructed using Illumina TruseqTM RNA sample prep Kit. The Illumina Novaseq 6000 platform was used for transcriptome sequencing, which generated a total of 254.5 Gb of sequence data. Upon filtering, the sequencing data to eliminate poor quality sequences, the sequences were mapped to the *Z. latifolia* genome using TopHat2 (http;//tophat.cbcb.umd.edu/) [72]. The Transcripts Per Million reads (TPM) algorithm was used for quantification of gene expression. The heatmap of the expression patterns of TCS genes was generated by Heml 1.0 (Heatmap Illustrator) software (CUCKOO, Wuhan, China). All the transcriptomic data have been deposited at GenBank under accession PRJNA664353.

### 3.6. RNA Isolation and Real-Time PCR

Total RNA was extracted using an RNAprep Pure Plant Plus Kit (polysaccharides and polyphenolics rich; TIANGEN, Beijing, China). The first cDNA strand was synthesized with 2 μg of total RNA by using a FastKing RT Kit (with gDNase) (TIANGEN, Beijing, China), as per the manufacturer’s instructions. qRT-PCR was performed with the CFX96 Touch C1000 Real-Time PCR System (Bio-Rad, Hercules, CA, USA) by using specific primers designed by Primer 5 Software (Table S3). Three biological and three technical replicates for each sample were performed with 20 µL of reaction volume using the SYBR^®^ Green Realtime PCR Master Mix (Toyobo, Osaka, Japan). The *ACTIN* gene was used as the housekeeping loading reference. The relative gene expression was calculated using the 2^−ΔΔCt^ method [73].

## 4. Conclusions

A role for cytokinin in gall formation was postulated. Here we observed a positive effect of cytokinin treatment on the gall formation in *Z. latifolia* in line with earlier reports [7,8], which raised the question of how cytokinin signaling regulates the gall formation. To address this problem, 69 two-component system genes, including 25 HK(L)s (21 HKs and 4 HKLs), 8 HPs (5 authentic and 3 pseudo-HPs), and 36 RRs (14 type A, 14 type B, 2 type C, and 6 pseudo-RRs), were identified in the *Z. latifolia* genome; the classification was supported by conserved motifs and domains, exon and intron structure, and phylogeny. Our phylogenetic analysis revealed the closest relationships between rice and *Z. latifolia* in seven species comparisons. Gene duplication events contributed to the TCS gene expansion in the *Z. latifolia* genome, and Ka/Ks analysis suggested that these duplicated gene pairs experienced strong positive selection during evolution.

We observed the activation of the expression of several of these TCS genes during gall formation, either in the very early stages or during the development of the gall. Among these, two genes encoding for RRA-type response regulators were also rapidly induced by cytokinin treatment. This transient upregulation of these RRA type was in line with RRA genes being a direct target of cytokinin signaling in other plant systems. Furthermore, the far-red light receptor phytochrome A was revealed to be a putative target.

In conclusion, these observations suggest an important role for cytokinin signaling in the process of gall formation and they highlight an important role for cytokinins in the ancient relationship between the endophyte *Ustilago esculenta* and its host *Z. latifolia.*

## Figures and Tables

**Table 1:** Histidine kinase/histidine-kinase-like (HK(L)) proteins in *Z. Latifolia*.

Gene ID	Gene Name	Character ^a^	Gene Family	Counterpart	Score (bits)	Identity (%)	Length (aa)	Molecular Weight (kDa)	Isoelectric Point (PI)	Number of TM	Subcellular localization ^b^
Zlat_10043954	ZlCHK1	HisKA, HATPase_c, CHASE, REC, TM	Cytokinin Receptor	AHK2	532	65	609	67.56	7.26	4	Endomembrane system
Zlat_10043955	ZlCHK2	HisKA, HATPase_c, REC	Cytokinin Receptor	AHK2	207	46	445	49.66	6.65	0	Nucleus
Zlat_10033100	ZlCHK3	HisKA, HATPase_c, REC	Cytokinin Receptor	AHK3	673	61	570	63.2	6.72	0	Nucleus
Zlat_10033475	ZlCHK4	HisKA, HATPase_c, CHASE, REC, TM	Cytokinin Receptor	AHK3	1045	63	861	95.89	8.55	1	Nucleus
Zlat_10002572	ZlCHK5	HisKA, HATPase_c, CHASE, REC, TM	Cytokinin Receptor	AHK4	746	68	907	99.75	6.02	2	Endomembrane system
Zlat_10008874	ZlCHK6	HisKA, HATPase_c, CHASE, REC, TM	Cytokinin Receptor	AHK4	202	72	430	48.69	5.58	1	Organelle membrane
Zlat_10009428	ZlCHK7	HisKA, HATPase_c, CHASE, REC, TM	Cytokinin Receptor	AHK4	620	69	618	69.42	5.27	1	Nucleus
Zlat_10007870	ZlCHK8	HisKA, HATPase_c, CHASE, REC, TM	Cytokinin Receptor	AHK4	335	55	716	78.44	5.41	1	Nucleus
Zlat_10005586	ZlCKI1	HisKA, HATPase_c, REC		CKI1	330	36	810	89.62	5.81	0	Nucleus
Zlat_10007295	ZlERS1	HisKA, HATPase_c, GAF, TM	Ethylene Receptor	ETR1	900	74	636	70.93	6.86	3	Endomembrane system
Zlat_10019946	ZlERS2	HisKA, HATPase_c, GAF	Ethylene Receptor	ETR1	380	65	314	34.48	5.29	0	Nucleus
Zlat_10028651	ZlERS3	HisKA, HATPase_c, GAF, TM	Ethylene Receptor	ETR1	879	75	635	70.79	6.82	3	Endomembrane system
Zlat_10043320	ZlETR1	HisKA, HATPase_c, GAF, REC, TM	Ethylene Receptor	ETR2	549	47	842	93.21	6.89	4	Plasma membrane
Zlat_10010084	ZlETR2	HisKA, HATPase_c, GAF, REC, TM	Ethylene Receptor	EIN4	669	51	759	85.01	6.21	3	Endomembrane system
Zlat_10043113	ZlETR3	HisKA, HATPase_c, GAF, REC, TM	Ethylene Receptor	EIN4	662	51	758	84.99	6.18	3	Plasma membrane
Zlat_10044950	ZlETR4	HisKA, HATPase_c, GAF, REC	Ethylene Receptor	EIN4	302	39	507	54.79	7.52	0	Nucleus
Zlat_10007862	ZlPHYA	HisKA, HATPase_c, GAF, PAS	Phytochrome	PHYA	1456	64	1128	125.1	5.78	0	Nucleus
Zlat_10002747	ZlPHYB	HisKA, HATPase_c, GAF, PAS	Phytochrome	PHYA	1439	63	1129	125.24	5.83	0	Nucleus
Zlat_10036932	ZlPHYC	HisKA, HATPase_c, GAF, PAS	Phytochrome	PHYB	1736	75	1190	130.73	5.72	0	Organelle membrane
Zlat_10005292	ZlPHYD	HisKA, HATPase_c, GAF, PAS	Phytochrome	PHYB	1710	75	1178	123.76	5.69	0	Nucleus
Zlat_10007618	ZlPHYE	HisKA, HATPase_c, GAF, PAS	Phytochrome	PHYC	1384	59	1137	125.65	5.62	0	Chloroplast
Zlat_10046362	ZlPDK1	HATPase_c	Pyruvate dehydrogenase kinase	PDK	530	74	363	40.77	6.08	0	Chloroplast
Zlat_10041425	ZlPDK2	HATPase_c	Pyruvate dehydrogenase kinase	PDK	521	72	363	40.73	6.33	0	Chloroplast
Zlat_10030552	ZlPDK3	HATPase_c	Pyruvate dehydrogenase kinase	PDK	551	76	363	40.91	6.68	0	Nucleus
Zlat_10018898	ZlPDK4	HATPase_c	Pyruvate dehydrogenase kinase	PDK	555	73	405	44.76	6.84	0	Chloroplast

^a^ Character indicates conserved histidine–kinase domain (HK), receiver domain (Rec), CHASE domain for cytokinin binding (CHASE), and chromophore-binding domain (PHY). ^b^ Subcellular localization predicted with BUSCA (Bologna Unified Subcellular Component Annotator).

**Table 2:** Histidine phosphotransfer proteins (HP) proteins in *Z. latifolia*.

Gene ID	Gene Name	Character ^a^	Gene Family	Counterpart	Score(bits)	Identity (%)	Length (aa)	Molecular Weight(kDa)	Isoelectric Point (PI)	Subcellular localization ^b^
Zlat_10020936	ZlHP1	HPt	HPt	AHP1	117	20	210	23.93	8.57	Organelle membrane
Zlat_10031305	ZlHP2	HPt	HPt	AHP1	115	50	146	16.63	5.28	Nucleus
Zlat_10002667	ZlHP3	pseudo-HPt	pseudo-HPt	AHP4	162	61	276	30.89	7.11	Extracellular space
Zlat_10013094	ZlHP4	HPt	HPt	AHP4	184	59	151	17.84	8.33	Nucleus
Zlat_10015279	ZlHP5	pseudo-HPt	pseudo-HPt	AHP4	187	59	158	18.03	8.2	Nucleus
Zlat_10032102	ZlHP6	pseudo-HPt	pseudo-HPt	AHP4	197	63	151	17.42	7.55	Nucleus
Zlat_10034774	ZlHP7	HPt	HPt	AHP5	125	46	149	16.78	4.71	Nucleus
Zlat_10034845	ZlHP8	HPt	HPt	AHP5	125	46	149	16.79	4.66	Nucleus

^a^ Character indicates whether the proteins possess a conserved His-containing phosphotransfer domain (HPt) or a pseudo-HPt lacking the His phosphorylation site. ^b^ Subcellular localization predicted with BUSCA (Bologna Unified Subcellular Component Annotator).

**Table 3:** Response regulator (RR) proteins in *Z. latifolia.*

Gene ID	Gene Name	Character ^a^	Gene Family	Counterpart	Score(bits)	Identity (%)	Length (aa)	Molecular Weight(kDa)	Isoelectric Point (PI)	Subcellular Localization ^b^
Zlat_10022933	ZlRRA1	REC	Type A	ARR3	103	61	167	16.23	5.71	Nucleus
Zlat_10026415	ZlRRA2	REC	Type A	ARR3	110	65	175	16.16	6.84	Chloroplast
Zlat_10028329	ZlRRA3	REC	Type A	ARR3	129	64	143	15.11	9.46	Nucleus
Zlat_10028974	ZlRRA4	REC	Type A	ARR3	129	64	137	14.75	6.59	Nucleus
Zlat_10030869	ZlRRA5	REC	Type A	ARR3	124	54	258	28.13	5.99	Extracellular space
Zlat_10032021	ZlRRA6	REC	Type A	ARR3	121	52	269	29.31	7.6	Plasma membrane
Zlat_10033406	ZlRRA7	REC	Type A	ARR6	152	64	165	17.89	9.1	Chloroplast outer membrane
Zlat_10001454	ZlRRA8	REC	Type A	ARR8	200	72	234	25.56	4.83	Nucleus
Zlat_10017478	ZlRRA9	REC	Type A	ARR8	99.4	59	187	21.08	6.81	Nucleus
Zlat_10003241	ZlRRA10	REC	Type A	ARR9	112	72	76	8.31	8.71	Extracellular space
Zlat_10016677	ZlRRA11	REC	Type A	ARR9	204	57	215	24.01	6.06	Nucleus
Zlat_10020468	ZlRRA12	REC	Type A	ARR9	137	81	176	19.42	6.74	Nucleus
Zlat_10029578	ZlRRA13	REC	Type A	ARR9	134	57	231	25.92	4.07	Extracellular space
Zlat_10044807	ZlRRA14	REC	Type A	ARR9	220	70	196	22.08	6.05	Nucleus
Zlat_10024295	ZlRRB1	REC, Myb	Type B	ARR1	352	57	689	73.95	5.99	Nucleus
Zlat_10041210	ZlRRB2	REC, Myb	Type B	ARR1	356	57	688	73.83	6.13	Nucleus
Zlat_10019278	ZlRRB3	REC, Myb	Type B	ARR2	186	39	353	40.14	7.07	Nucleus
Zlat_10010976	ZlRRB4	REC, Myb	Type B	ARR10	278	42	627	68.56	5.95	Nucleus
Zlat_10006425	ZlRRB5	REC, Myb	Type B	ARR11	128	62	179	20.47	4.81	Nucleus
Zlat_10007685	ZlRRB6	REC, Myb	Type B	ARR11	47.4	30	340	36.56	5.61	Cytoplasm
Zlat_10010099	ZlRRB7	REC, Myb	Type B	ARR11	328	50	583	65.13	5.04	Nucleus
Zlat_10033001	ZlRRB8	REC, Myb	Type B	ARR11	348	54	580	65.07	5.16	Nucleus
Zlat_10007027	ZlRRB9	REC, Myb	Type B	ARR12	321	49	707	76.01	6.25	Nucleus
Zlat_10018529	ZlRRB10	REC, Myb	Type B	ARR12	343	55	694	76.02	5.87	Nucleus
Zlat_10018563	ZlRRB11	REC, Myb	Type B	ARR12	322	66	693	75.34	6.2	Nucleus
Zlat_10022449	ZlRRB12	REC, Myb	Type B	ARR12	336	59	621	68.29	5.88	Nucleus
Zlat_10028769	ZlRRB13	REC, Myb	Type B	ARR12	348	60	626	68.84	5.8	Nucleus
Zlat_10041971	ZlRRB14	REC, Myb	Type B	ARR14	106	46	299	31.15	6.61	Nucleus
Zlat_10027036	ZlRRC1	REC	Type C	ARR24	66.6	40	128	13.85	5.69	Nucleus
Zlat_10029496	ZlRRC2	REC	Type C	ARR24	67.8	41	129	13.8	5.68	Nucleus
Zlat_10006383	ZlPRR1	Pseudo-REC, CCT	Pseudo	APRR1	306	39	518	57.68	6.34	Nucleus
Zlat_10008561	ZlPRR2	Pseudo-REC, CCT	Pseudo	APRR7	318	41	764	82.49	8.52	Chloroplast outer membrane
Zlat_10024918	ZlPRR3	Pseudo-REC, CCT	Pseudo	APRR7	347	40	742	81.17	6.23	Nucleus
Zlat_10017908	ZlPRR4	Pseudo-REC, CCT	Pseudo	APRR5	228	39	629	70.06	6.78	Nucleus
Zlat_10023948	ZlPRR5	Pseudo-REC, CCT	Pseudo	APRR5	212	53	684	74.99	8.21	Nucleus
Zlat_10035513	ZlPRR6	Pseudo-REC, CCT	Pseudo	APRR5	215	54	682	75.18	8.44	Nucleus

^a^ Character indicates receiver domain (REC), pseudo-receiver domain (pseudo-REC) lacking the conserved D, Myb-like domain (Myb), plant-specific CCT motif found in clock proteins. ^b^ Subcellular localization predicted with BUSCA (Bologna Unified Subcellular Component Annotator).

## Supplementary Materials

The following are available online at https://www.mdpi.com/2223-7747/9/11/1409/s1, Figure S1: Amino acid sequence alignment of ZlHK(L)s in *Z. Latifolia*; Figure S2: Phylogenetic relationship, gene structures, and conserved motif of all HK(L) genes in *Z. latifolia*; Figure S3: Phylogenetic relationship, gene structures, and conserved motif of the HP family members in *Z. Latifolia*; Figure S4: Amino acid sequence alignment of ZlHP proteins in *Z. latifolia*; Figure S5: Phylogenetic relationship, gene structures, and conserved motif of RR genes in *Z. latifolia*; Figure S6: The validation of TCS gene expression by qPCR; Table S1: Summary of the two-component system (TCS) gene numbers identified in plants; Table S2: The Ka/Ks ratios of duplicated TCS genes in *Z. latifolia*; Table S3: The primer sequences.
