# Supplementary material for: Genome-Wide Characterization and Expression of Two-Component System Genes in Cytokinin-Regulated Gall Formation in Zizania latifolia
Source: Plants (Basel). 2020 Oct 22;9(11):1409. doi: 10.3390/plants9111409 (PMC7690396; doi:10.3390/plants9111409)
Supplement: Supplementary file 1 [file plants-09-01409-s001.zip › plants-958153-supplementary-forconversion/manuscript and supplement/Supplementary Figures.docx]

**Supplementary Figures**

A


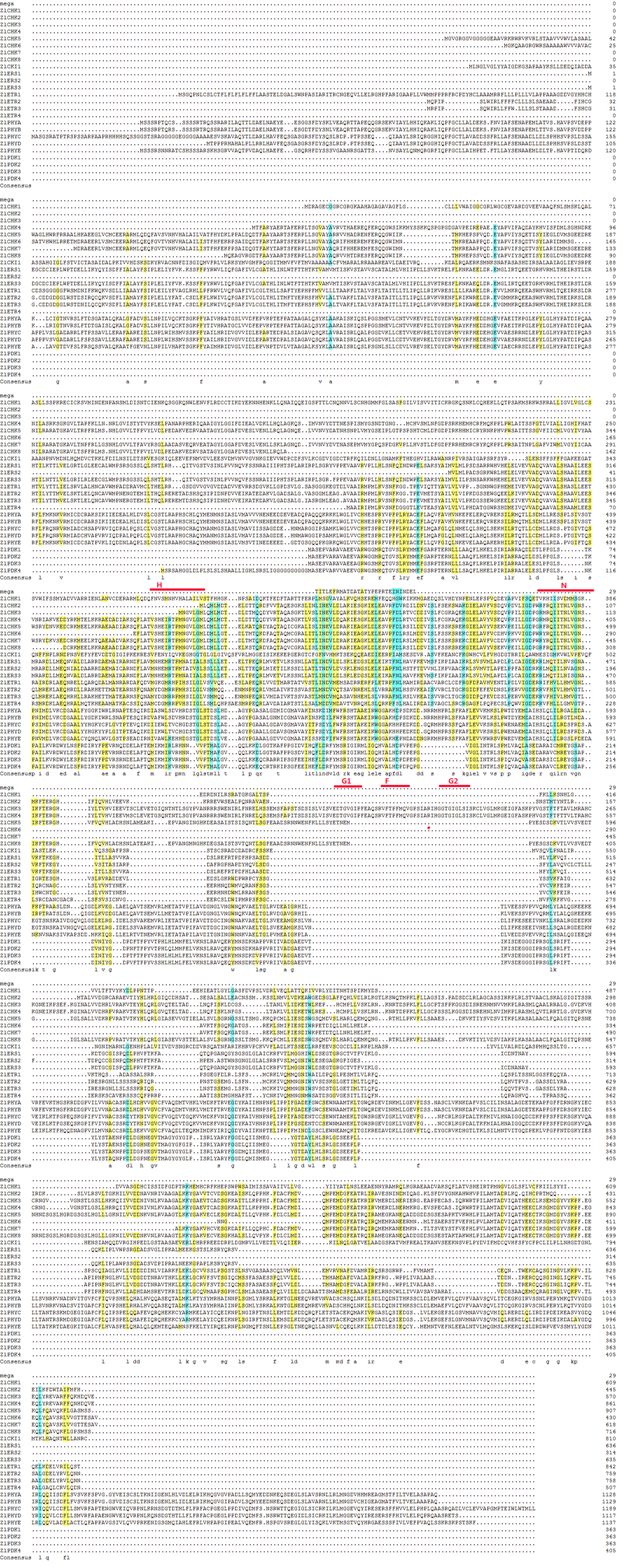


B


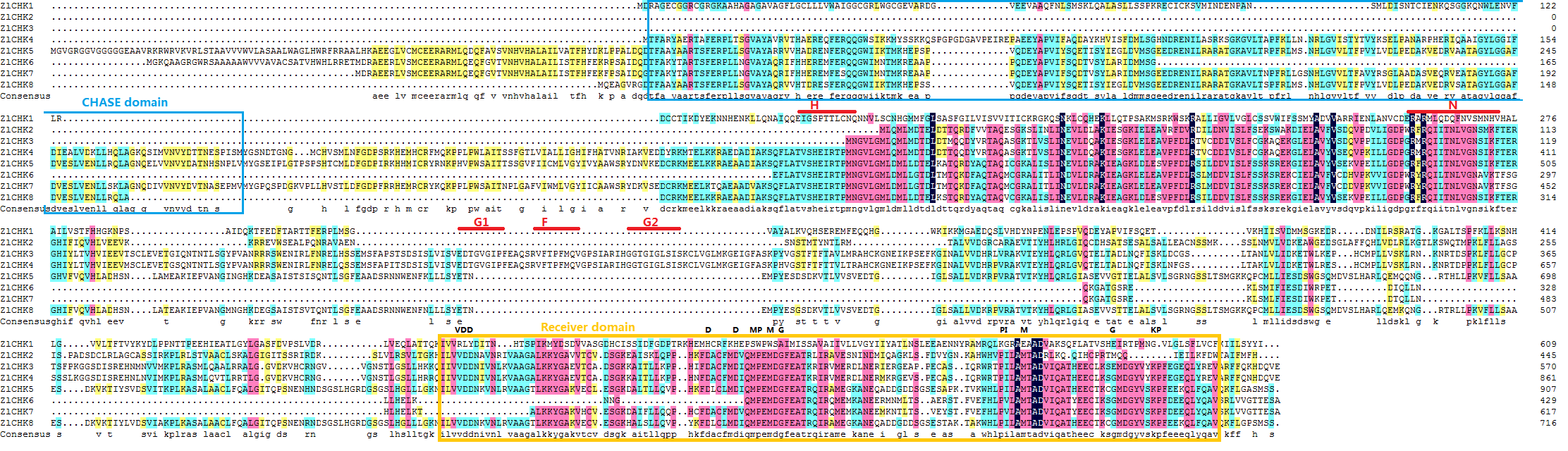


**Figure S1.** Amino acid sequence alignment of ZlHK(L)s in *Z. latifolia*. Histidine kinase (like) (A), receiver and cyclase/histidine kinase-associated sensory extracellular (CHASE) domains (B) from ZlHK(L) proteins in *Z. latifolia* were aligned by the Clustal X program. The conserved motifs have been marked.

**
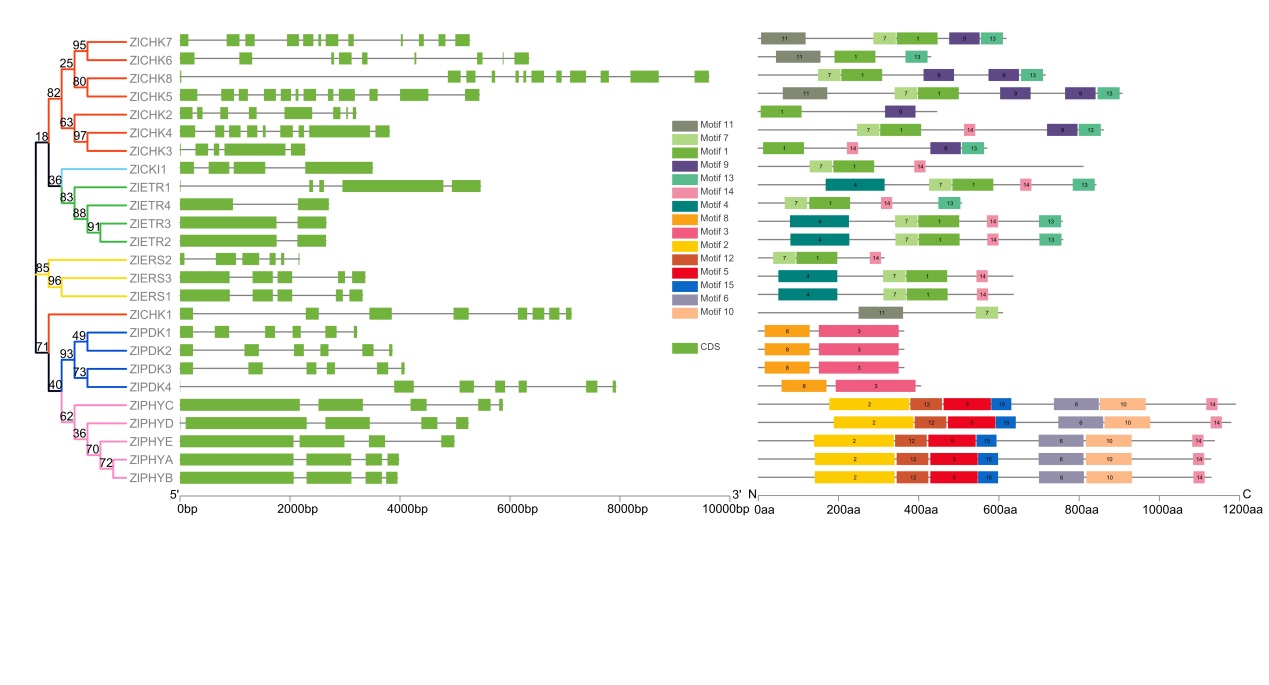
**

**Figure S2.** Phylogenetic relationship, gene structures, and conserved motif of all HK(L) genes in *Z. latifolia*. The green boxes indicate the exons, and lines indicate the introns. The different colored boxes represent different motifs in the corresponding position of each ZlHK(L) proteins.


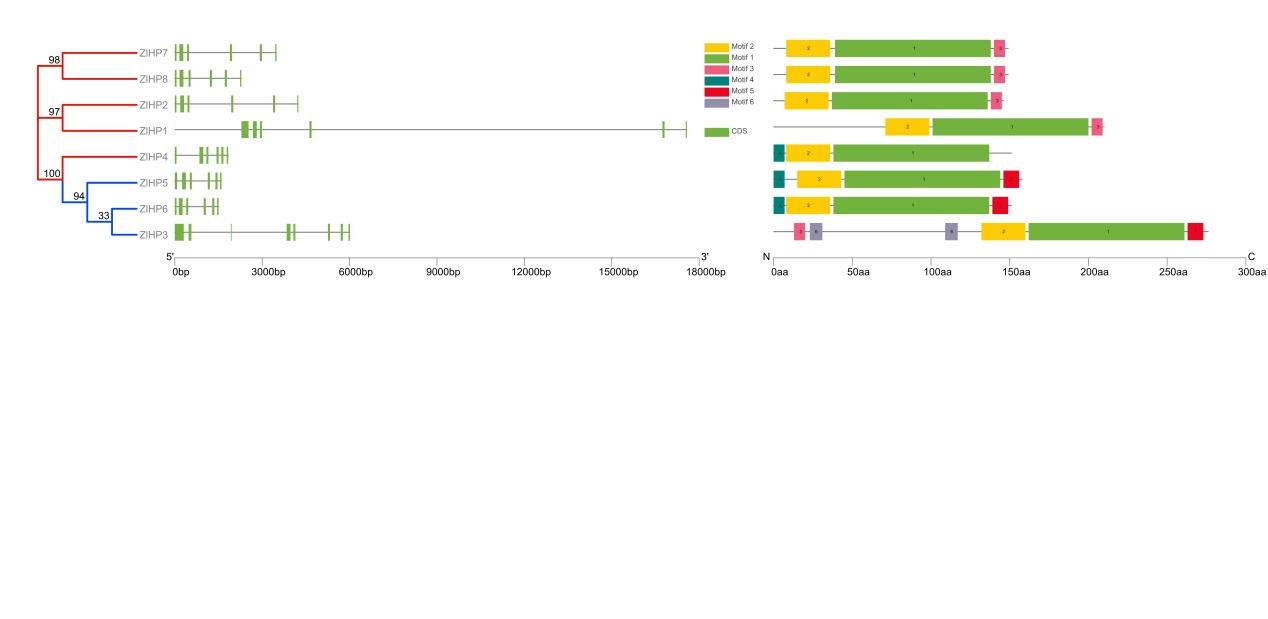


**Figure S3.** Phylogenetic relationship, gene structures, and conserved motif of the HP family members in *Z. latifolia.*


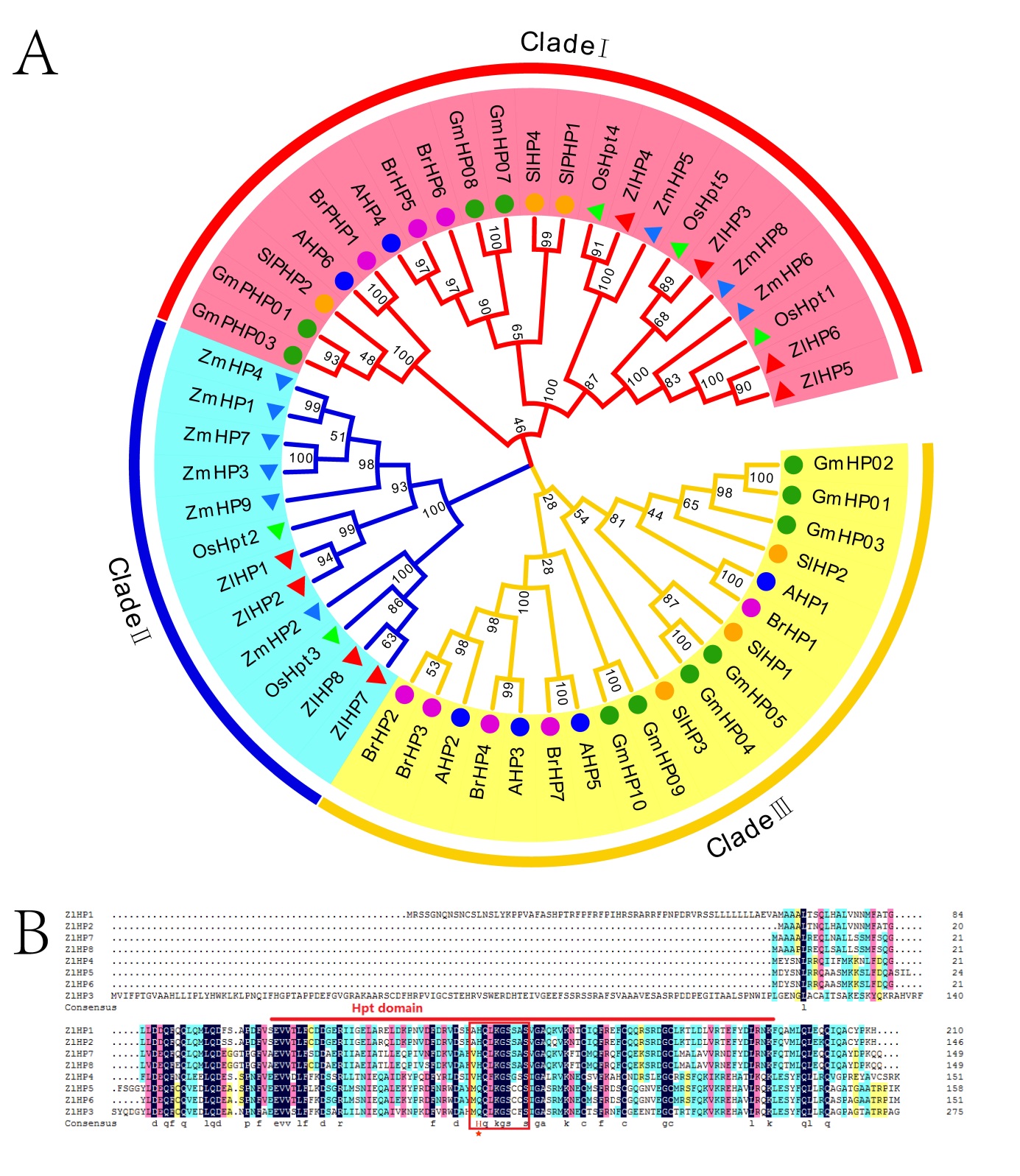


**Figure S4.** Amino acid sequence alignment of ZlHP proteins in *Z. latifolia*. Sequences were aligned by the Clustal X program. The Hpt domain has been highlighted by red line. The conserved XHQXKGSSXS motif was also marked above the alignment.


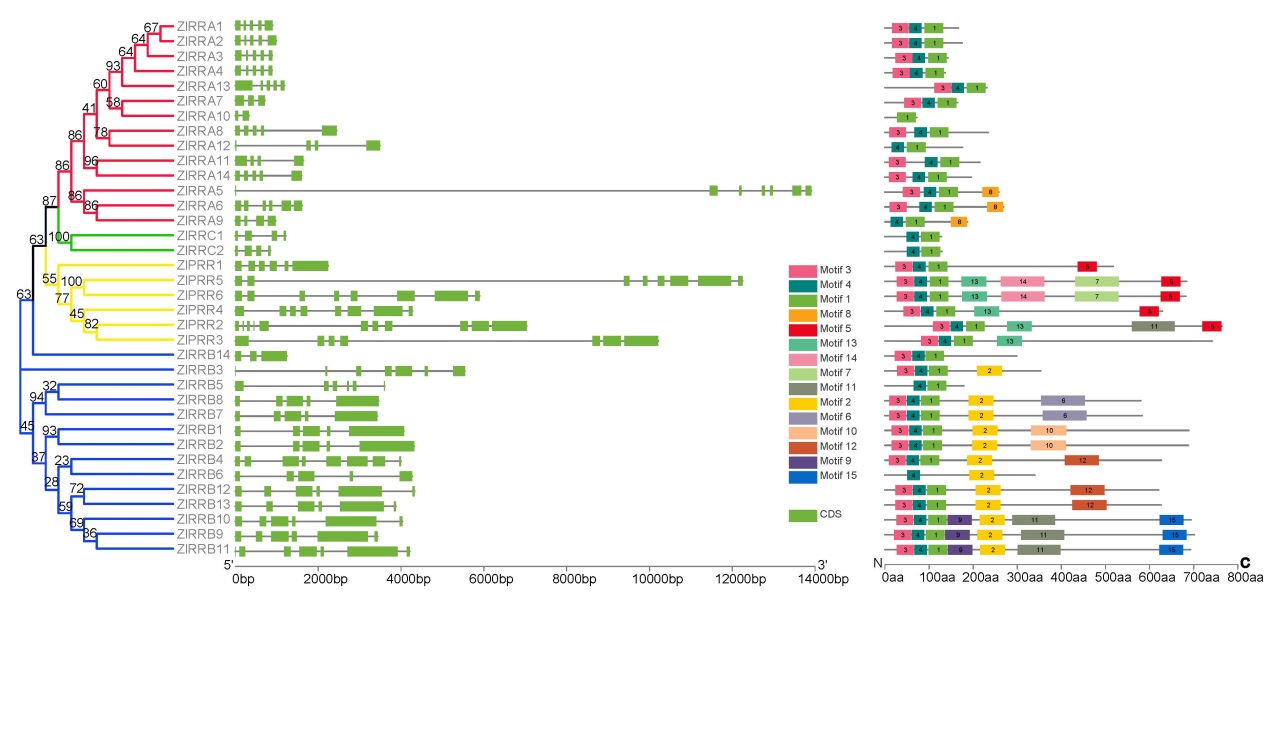


**Figure S5.** Phylogenetic relationship, gene structures, and conserved motif of RR genes in *Z. latifolia.*


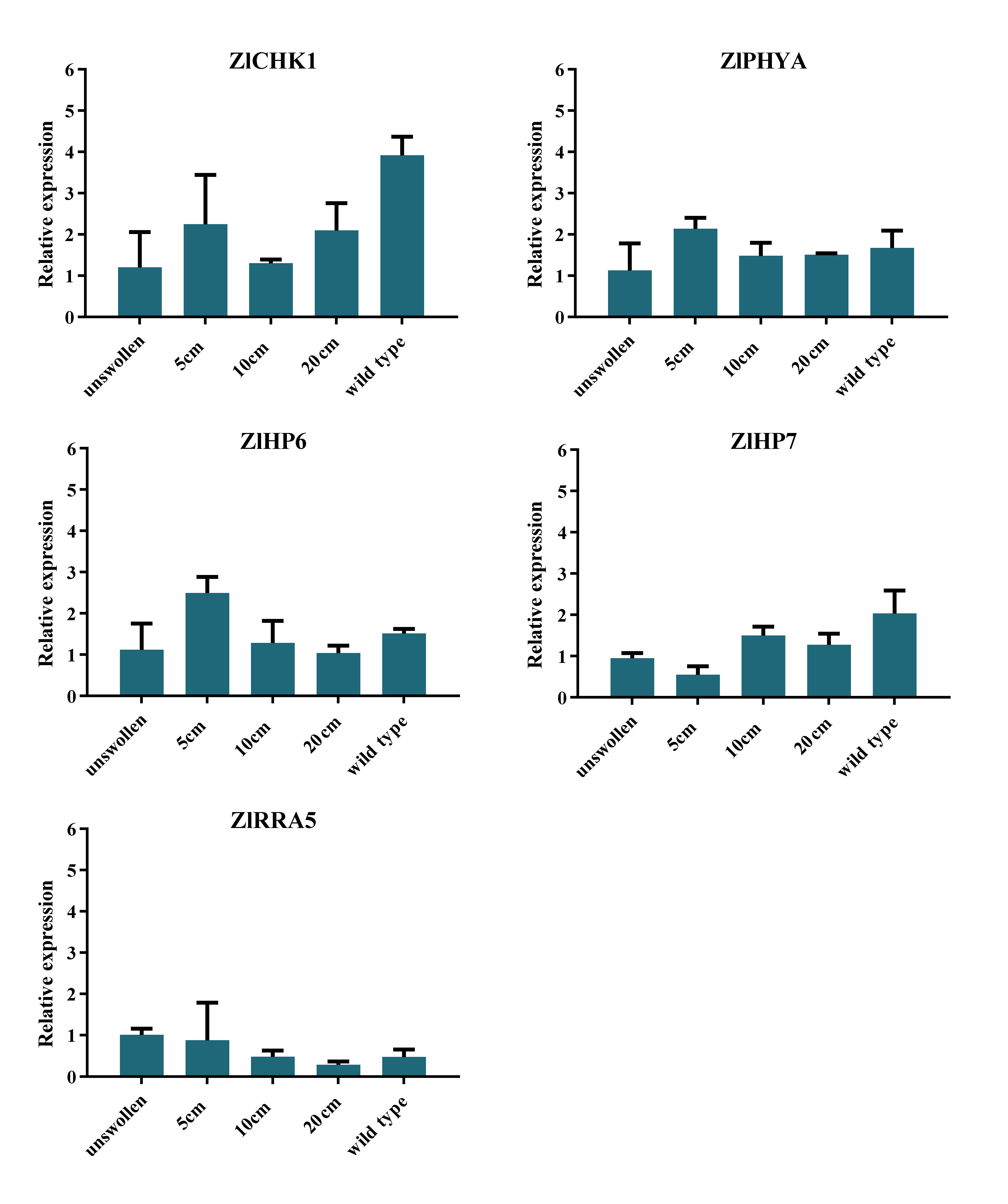


**Figure S6.** The validation of TCS genes expression by qPCR (Wild-type sample is from different genetic background, so the unswollen sample was used as normalisation standard.)
