# Supplementary material for: Genome-Wide Characterization and Expression of Two-Component System Genes in Cytokinin-Regulated Gall Formation in Zizania latifolia
Source: Plants (Basel). 2020 Oct 22;9(11):1409. doi: 10.3390/plants9111409 (PMC7690396; doi:10.3390/plants9111409)
Supplement: Supplementary file 1 [file plants-09-01409-s001.zip › plants-958153-supplementary-forconversion/manuscript and supplement/Supplementary Materials.docx]

**Supplementary Figures** **and Tables**

**Figure S1.** Amino acid sequence alignment of ZlHK(L)s in *Zizania Latifolia*.

**Figure S2.** Phylogenetic relationship, gene structures, and conserved motif of all HK(L) genes in *Zizania Latifolia.*

**Figure S3.** Phylogenetic relationship, gene structures, and conserved motif of the HP family members in *Zizania Latifolia*.

**Figure S4.** Amino acid sequence alignment of ZlHP proteins in *Zizania Latifolia*.

**Figure S5.** Phylogenetic relationship, gene structures, and conserved motif of RR genes in *Zizania Latifolia*.

**Figure S6.** The validation of TCS genes expression by qPCR

**Table S1.** Summary of the two-component system (TCS) gene numbers identified in plants.

**Table S2.** The Ka/Ks ratios of duplicated TCS genes in Zizania Latifolia.

**Table S3.** primers in our study

**Table S4.** The validation of TCS genes expression by qPCR
